# Target repositional accuracy and PTV margin verification using three‐dimensional cone‐beam computed tomography (CBCT) in stereotactic body radiotherapy (SBRT) of lung cancers

**DOI:** 10.1120/jacmp.v13i2.3708

**Published:** 2012-03-08

**Authors:** Lu Wang, Steven Feigenberg, Jiajin Fan, Lihui Jin, Aruna Turaka, Lili Chen, C‐M Charlie Ma

**Affiliations:** ^1^ Fox Chase Cancer Center Philadelphia PA 19111 USA

**Keywords:** target localization, cone‐beam CT, lung SBRT

## Abstract

The purpose of this study was to assess target repositional accuracy with respect to the bony structures using daily CBCT, and to validate the planning target volume (PTV) margin used in the lung SBRT. All patients underwent 4D CT scanning in preparation for lung SBRT. The internal target volume (ITV) was outlined from the reconstructed 4D data using the maximum‐intensity projection (MIP) algorithm. A 6 mm margin was added to the ITV to create the PTV. Conformal treatment planning was performed on the helical images, to which the MIP images were fused. Prior to each treatment, CBCT was taken after a patient was set up in the treatment position. The CBCT images were fused with the simulation CT based on the bony anatomy, in order to derive setup errors and separate them from the tumor repositional errors. The treating physician then checked and modified the alignment based on target relocalization within the PTV. The shifts determined in such a method were recorded and the subtractions of these shifts with respect to the corresponding setup errors were defined as the target relocalization accuracy. Our study of 36 consecutive patients, treating 38 targets for a total of 153 fractions shows that, after setup error correction, the target repositional accuracy followed a normal distribution with the mean values close to 0 in all directions, and standard deviations of 0.25 cm in A–P, 0.24 cm in Lat, and 0.28 cm in S–I directions, respectively. The probability of having the shifts ≥0.6cm is less than 0.8% in A–P, 0.6% in Lat, and 1.7 % in S‐I directions. For the patient population studied, the target centroid position relative to the bony structures changed minimally from day to day. This demonstrated that the PTV margin that is designed on the MIP image‐based ITV was adequate for lung SBRT.

PACS number: 87.53.Ly

## I. INTRODUCTION

Organ motion is an important consideration during radiotherapy treatment planning and delivery for patients with lung cancer.^(^ ^1^ ^–^ ^7^ ^)^ When free‐breathing computed tomography (CT) scans are used for planning, variable geometric errors can arise with respect to the position, shape, and volume of the gross tumor volume (GTV) and surrounding normal structures.^(^ ^1^ ^,^ ^8^ ^)^ With the improvement in imaging technology, four‐dimensional CT (4D CT) has been developed to overcome the difficulties which occur during imaging acquisition due to respiratory motion. Using 4D CT technology, volumetric images can be acquired at various times during the respiratory cycle; the time‐resolved imaging techniques eliminate respiratory‐induced artifacts and allow tumor motion to be characterized specifically.^(^ ^9^ ^,^ ^10^ ^)^ This specific information can aid treatment planners in designing the patient‐specific planning target volume (PTV), which could further improve targeting and planning accuracy,^(^ ^11^ ^–^ ^14^ ^)^ as well as potentially reduce doses to surrounding normal structures, thus decreasing side effects. This approach of individualized margins based on patient‐specific tumor motion has been proposed for high‐dose hypofractionated SBRT.^(^ ^5^ ^,^ ^13^ ^–^ ^19^ ^)^

In order to efficiently delineate tumor motion in all of the respiratory phases for the target volume definition, a maximum intensity projection (MIP) algorithm was proposed.^(20)^ This method used the highest data value encountered along the viewing ray for each pixel of volumetric data, giving rise to a full intensity display of the brightest object along each ray of the projection image.^(21)^ Several studies have shown that MIP‐reconstructed CT accurately reflects the range of target motion for regular target motion and the use of MIP for internal target volume (ITV) definition in 4D CT scans for lung cancer is fairly reliable.^(^ ^20^ ^,^ ^22^ ^,^ ^23^ ^)^ Therefore, according to ICRU Report 62,^(24)^ a PTV should be defined with a safety margin that accounts for daily setup error only, given the fact that tumor motion has been accounted for in the ITV (=CTV) definition.

However, the tumor motion pattern may change from day to day^(^ ^25^ ^–^ ^30^ ^)^ and even during treatment delivery.^(^ ^31^ ^,^ ^32^ ^)^ This implies that a ITV design based on one set of 4D CT (during simulation) may not be accurate for treatment planning.^(33)^ Moreover, the PTV margin designed to account for setup error only may not be enough to account for the change of the centroid position of the ITV during the treatment course. Several studies have used 4D CT or respiration‐correlated CT images to measure intra‐ and interfractional tumor motion.^(^ ^26^ ^,^ ^28^ ^,^ ^34^ ^)^ That research focused mainly on measuring the motion magnitude of the gross tumor volume (GTV) from its baseline position (e.g., zero respiration phase) due to respiration. Only a few investigators have studied tumor repositional inaccuracies with respect to the patient's bony structures during treatment delivery.^(^ ^30^ ^,^ ^35^ ^,^ ^36^ ^)^ However, studies that direct use of this tumor repositional inaccuracy data for PTV margin verification is sparse^(^ ^36^ ^,^ ^37^ ^)^ not to mention that, in our situation, the PTV is generated from an ITV delineated from MIP‐reconstructed 4D CT images. From a clinical point of view, the positional change of the ITV with respect to the patient's bony structures and the PTV margin are most relevant to the coverage of disease and requires further study.

In this work, the overall target volume position change with respect to the bony structures using cone‐beam computed tomography (CBCT) was assessed and evaluated to ensure that the PTV margin used in the lung stereotactic body radiotherapy (SBRT) was appropriate in the treatment room. Gantry‐mounted and flat‐panel–based kV cone‐beam imaging is a new imaging modality developed recently which provides a superior soft tissue contrast for 3D image‐guided radiation therapy.^(^ ^38^ ^,^ ^39^ ^)^ Due to the time required to obtain the cone‐beam CT image dataset, the imaged target should have gone through several respiratory cycles such that the image contains not only the target but this motion also.^(39)^ Song et al.^(40)^ demonstrated that cone‐beam CT is equivalent to a subsequently reconstructed 4D CT MIP image set. Because of this equivalence, we could identify and compare the ITV position in the daily cone‐beam CT versus what appeared on the simulation 4D CT. Through that comparison, we could determine daily target repositional accuracy and, thus, we could determine whether the PTV margin was appropriate for covering the daily target centroid position change.

## II. MATERIALS AND METHODS

The stereotactic body radiotherapy program at Fox Chase Cancer Center started in 2004. Patients were treated using patient‐specific ITV while adding a symmetric 5.0–6.0 mm margin to make a PTV. The standard dose fraction was 48 Gy in four fractions, with dose typically prescribed to the 90% isodose surface, which covered at least 95% of the PTV. Central tumors were treated with a lower dose per fraction (60 Gy in eight fractions or 50 Gy in 10 fractions). This study evaluated 36 consecutive patients who received SBRT treatment in our institution in 2009. Among the 36 patients, two patients had 2 targets and a total of 153 treatment fractions were analyzed. Patient ages ranged from 54 to 89, with a median age of 71. There were 15 targets in the right upper lobe (RUL), six targets in the right lower lobe (RLL), one target in the middle of the right lobe, nine targets in the left upper lobe (LUL), and six targets in the left lower lobe (LLL).

### A. Image acquisition and target definition

For all lung SBRT patients, a GE LightSpeed CT scanner (GE Healthcare, Waukesha, WI) and its 4D CT image processing and reconstruction software were used to collect 4D CT images. Patients were scanned in the head‐first supine position with their arms above their head. Immobilization was established with an inflatable vacuum cradle. All planning CT images were obtained at 2.5 mm slice thickness. For each patient, transverse slices were acquired both in the helical mode and cine mode using a four‐slice per rotation (0.5 sec/rotation) setting. During acquisition of the CT images, the respiratory signal was recorded with the real‐time position management (RPM) respiratory gating system (Varian Medical System, Palo Alto, CA) and synchronized with the CT data acquisition. In this way, each image was tagged according to the phase of the respiratory cycle in which it was acquired. The CT scanning technologists usually will track patient breathing and coach the patient in order to obtain a regular breathing pattern. Both helical images for free breathing and the 4D CT dataset were then sent to the Advantage Workstation (General Electric Company, Waukesha, WI) using the Advantage 4D CT software. 4D CT images were grouped and sorted into their respective phases. A reconstructed dataset was generated using the maximum‐intensity projection (MIP) protocol. MIPs create a 3D CT scan which represents the greatest voxel intensity values throughout the 4D CT dataset.

Before contouring, the MIP image set was fused to the helical image set, as the latter one was used for treatment planning. The ITV was contoured by the treating physician directly on the MIP images, using the lung window. Only the visualized solid tumor (with its motion) component was targeted. Spicules were not contoured. The CTV was defined as the ITV with no additional margin. Theoretically, the PTV margin should account for setup uncertainties and internal target motion. In our approach, since daily cone‐beam CT has been used for imaging guidance, setup uncertainty can be minimized. Nevertheless, there are uncertainties inherent to the imaging software and imaging fusion process. One such uncertainty is associated with the resolution of cone‐beam CT^(26)^ which, in our situation, is approximately 1 mm in the axial direction, and 3 mm in the longitudinal direction. The finite resolution limits the ability to reliably identify reference bony landmarks in the images. We named these the residual setup errors. In addition, the ITV delineated from the MIP images, although patient‐specific, may not account for daily changes in the tumor motion pattern. Thus, in order to safely account for both the residual setup errors and possible change of the tumor motion pattern, we have used 5–6mm as the PTV margin, which was also recommended by another group.^(31)^ One of the goals in the study is to determine whether this margin is indeed appropriate for this purpose.

### B. Treatment planning

The fused helical images with contoured structures were transferred to the Eclipse (Varian Medical System, Palo Alto, CA) treatment planning system (TPS). Conformal treatment planning was performed generally using 7–9 coplanar beams. Inhomogeneity corrections based on the equivalent path length method were employed. The isocenter was always placed at the center of the target. Beam shaping was achieved by using a Varian 5 mm leaf width Millennium multileaf collimator (Varian Medical System, Palo Alto, CA) and the block edge margins were 4 mm in the axial direction and 7 mm in the superior and inferior direction. The beam angles were selected so as to minimize dose to the lung, esophagus, heart, and spinal cord. Generally, 85%–90% isodose lines covered the entire PTV, and the dose was prescribed mostly to the 90% isodose line, making the 100% dose coverage at least 95% to 97% of the PTV.

### C. Image guidance using cone‐beam CT

**Figure 1:**
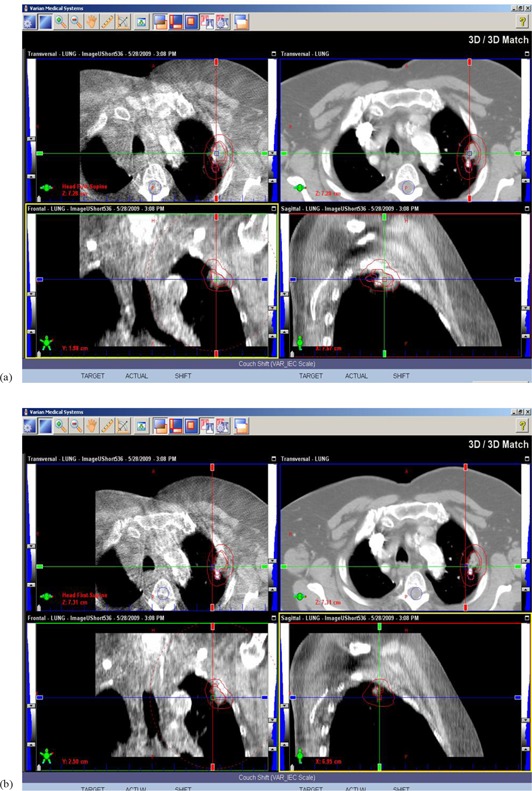
An example of image fusion between daily cone‐beam images and the simulation images: (a) after bony alignment but before soft tissue alignment; (b) after soft tissue alignment.

Before a treatment, each patient was set up on the Varian manufactured couch and immobilized in his/her vacuum cradle which was made during the CT simulation. The on‐board kV imaging system installed on the Varian Trilogy machine was used for 3D cone beam CT acquisition after the isocenter was located, either based on the tattoo marks or according to the shift information documented during the planning procedure. Therapists at the machine performed image fusion between the cone‐beam CT and the planning CT based purely on the patient's bony structure, which is usually the vertebral body of the patient. The shifts derived based on bony landmark alignment were recorded. The treating physician was informed and was responsible for checking the alignment. The treating physician would not only check the fusion accuracy, but also check to see if the visualized target volume was within the PTV. If it was not, the treating physician would make further adjustments in image fusion based on soft tissue alignment; that is, aligning the CBCT‐based target volume within the center of the PTV. The shifts based on soft tissue alignment were recorded for this study. However, for the actual treatment, the treating physician had the option of whether or not to apply the second shifts, based on personal judgment. Figure 1 shows an example of image fusion between daily cone‐beam images and the simulation images. Figure 1(a) shows the alignment after bony match but before soft tissue match; Fig. 1(b) shows the alignment after soft tissue match.

In this work, shifts made based on bony structure alignment are defined as the patient setup error and the subtractions of the final shifts (based on soft tissue alignment) to the setup errors are defined as the target repositional uncertainty with respect to the patient's bony structure. Given the target locations, we are able to examine whether the setup error and the changes in tumor average position are correlated with the target locations. In addition, by studying the target repositional accuracy, we can determine whether the tumor centroid position relative to the bony structures changes from day to day and, furthermore, whether the PTV margin is enough to account for daily target relocalization.

## III. RESULTS

**Figure 2:**
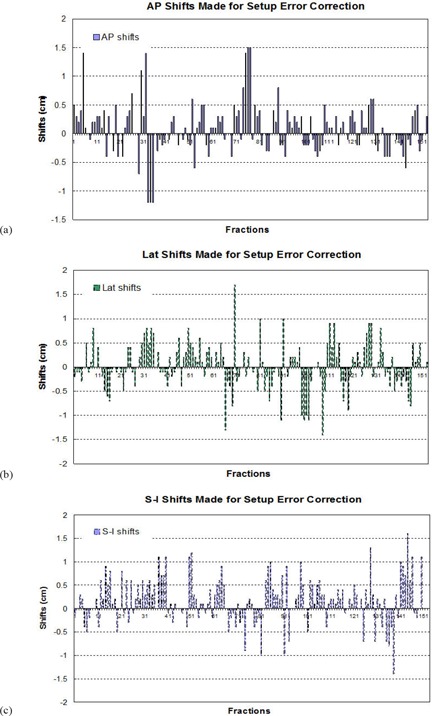
Setup errors in A–P (a), Lat ((b), and S–I (c) directions for 153 fractions. Data were derived based on bony landmark alignments.

**Figure 3:**
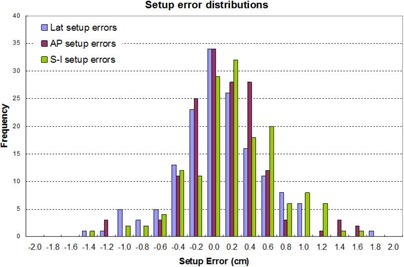
The distributions of the setup errors of all three major directions.

The following data were derived from the treatment records of 36 consecutive patients, 38 targets, and 153 treatment fractions. Figures 2(a), 2(b), and 2(c) show the setup errors in A–P, Lat, and S–I directions for 153 fractions, respectively. The maximum setup error is as large as 15 mm in A–P direction, 17 mm in Lat direction, and 16 mm in S–I direction. However, the average setup errors are 0.85 mm with a standard deviation of 4.3 mm in A–P, 0.13mm±4.9mm in Lat, and 1.7mm±4.9mm in S–I directions. Figure 3 shows the distributions of the setup errors. It is seen that the distributions follow bell‐shaped distributions with the mean values and standard deviations given above. The deviation from a standard normal distribution could be due to limited data sampling. The nonzero mean values imply that there were systematic errors in the setup process, typically due to the use of patient tattoos for setup, and skin sag makes this approach unreliable for some patients with skin laxity. From the distribution, we could calculate the probability of having setup errors less than 6 mm (which is the margin used for PTV design) and found it to be 88.4% in A–P, 88.5% in Lat, and 80.8% in S–I directions, respectively.

**Figure 4:**
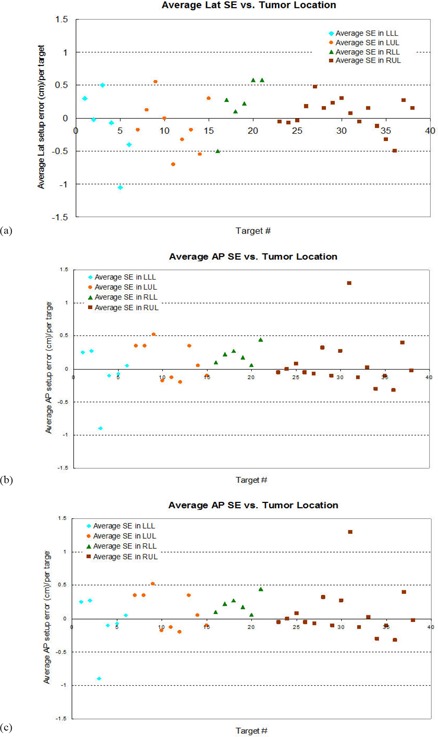
Average setup errors per patient/per target sorted based on the tumor locations (LLL, LUL, RLL, and RUL): (a), (b), and (c) correspond to the setup errors in the Lat, A–P, and S–I directions, respectively.

In order to determine if the setup errors have any correlation with the tumor location, we first calculated average setup errors for each patient (or each target) over the fractions along the Lat, A–P, and S–I directions, respectively, and sorted the data based on the tumor location. Figure 4 shows these errors. Figs. 4(a), (b), and (c) correspond to Lat, A–P, and S–I directions, respectively, for each patient/each target in four different location (LLL, LUL, RLL, RUL) groups. The averages among each group were also derived and have been listed in Table 1, along with the corresponding standard deviation. It is seen that for the tumor in the left lower lobe (LLL), the setup has greater random uncertainties (standard deviations) in the Lat (5.5 mm) and A–P (4.3 mm) directions, while for the tumors in the right upper lobe, the largest random error appears for the S–I direction. The random setup errors are similar for the tumor located in the LUL and RLL along the Lat and S–I directions, with similar values of 3.9 mm and 4.0 mm.

**Table 1:** The group means and standard deviations of the average setup errors per target in four different locations.

*Locations*	*Lat (mm)*	*A–P (mm)*	*S–I (mm)*
LLL	−1.25±5.50	−0.83±4.30	1.58±1.42
LUL	−1.06±3.98	1.14±2.80	2.22±3.79
RLL	2.08±3.98	2.15±1.40	2.58±3.89
RUL	0.50±2.43	0.78±3.81	1.12±4.20

**Figure 5:**
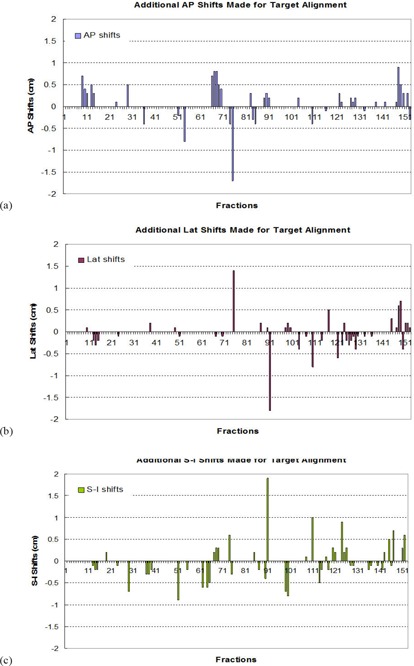
Target relocalization uncertainties along the three major directions with respect to the patient bony structure, which are defined as the additional shifts that the physician has made in order to align the target within the PTV.

**Figure 6:**
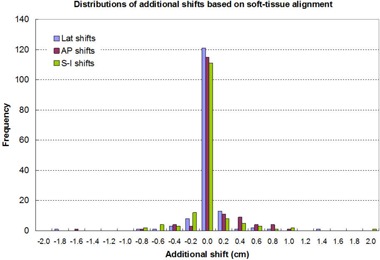
Distributions of the target relocalization uncertainties along the three major directions.

Figure 5 shows target repositional uncertainties with respect to the patient's bony structure, which are defined as the additional shifts that the physician has made in order to align the target within the PTV. Figure 6 presents the distributions of these inaccuracies along the three major directions. The distributions follow a standard normal distribution with the mean values around 0 in all directions and corresponding standard deviations of 2.5 mm in the A–P, 2.4 mm in the Lat, and 2.8 mm in the S–I directions, respectively. Although the target repositional uncertainty could be large (maximum at 18 mm) individually, the probability of having the uncertainty ≥6mm is ≤0.8% in the A–P, 0.6% in the Lat, and 1.7% in the S–I directions. This suggests that the fractions that do not require additional shifts are 99.2% in the A–P, 99.4% in the Lat, and 98.3% in the S–I directions.

**Figure 7:**
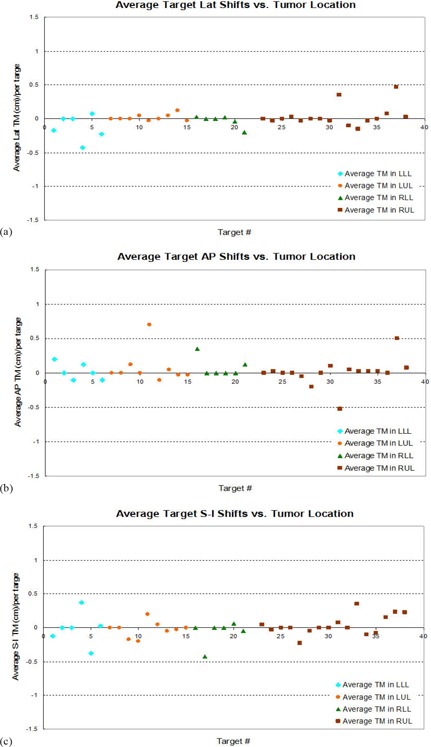
Average target relocalization uncertainties per patient/per target sorted based on the tumor locations (LLL, LUL, RLL, and RUL): (a), (b), and (c) correspond to the target uncertainties in the Lat, A–P, and S–I directions, respectively.

For the target repositional uncertainty, we also derived the averages per each patient or each target over the treatment fractions and sorted them according to location. This is to investigate whether the change of the tumor centroid position has anything to do with the tumor location. Figure 7 shows average changes in the target centroid position (i.e., average target relocalization accuracy) per each patient or each target versus the tumor location in the Lat (Fig. 7(a)), the A–P (Fig. 7(b)), and the S–I (Fig. 7(c)) directions, respectively. The averages among each group were also derived and are listed in the Table 2, along with the corresponding standard deviation. It is seen that the largest random errors (standard deviations) in the range of 2.4 mm are associated with the tumor motion in the S–I direction for the tumor in the LLL, and in the A–P directions for the tumor in the LUL. Tumors in the RUL tend to have a larger change in their centroid positions in the A–P direction.

**Table 2:** The group means and standard deviations of the average changes of target centroid position per target in four different locations.

*Locations*	*Lat (mm)*	*A–P (mm)*	*S–I (mm)*
LLL	−1.25±1.86	0.20±1.21	−0.17±2.43
LUL	0.19±0.48	0.81±2.41	−0.22±1.18
RLL	−0.33±0.33	0.79±1.42	−0.69±1.78
RUL	0.37±1.55	0.03±1.98	0.38±1.42

## IV. DISCUSSION

In this study, we used patient bony structures as a reference for determining the interfractional changes of the target centroid position. We found that, although individual large changes of the target centroid position were found, the average target centroid position (or overall target volume) relative to the bony structure changes is ≤1mm for the patient population studied. These results are similar to those obtained by Matsugi et al.,^(26)^ where they separately investigated the GTV centroid displacements between end‐exhalation and end‐inhalation for upper and lower lobe sited tumors, using three sets of 4D CT images taken during the course of treatment. They stated that the interfractional variation of the GTV motion range and centroid position did not vary significantly. Unlike their study, we used four to five sets of localization cone‐beam CT images for each patient while patients were in their treatment position. The image fusion between simulation CT and cone‐beam CT was performed by experienced therapists and checked by the treating physicians. Although this is not as precise as it appears, it is clinically relevant. The fusion accuracy is subjective to the magnitude of image resolution. For cone‐beam CT, the resolutions are about 1 mm on the axial images and 3 mm between the slices. Half of these values represent the uncertainties embedded in the image fusion.^(41)^

We also looked into cases in which a large change (≥6mm) of the target centroid position existed and found that, for two patients, the changes ≥6mm appeared for two of the four fractions; and for another patient, the changes ≥6mm appeared for all three fractions recorded. For some other patients, changes ≥6mm only appeared once during the entire treatment. For those patients who constantly presented a larger change of the target centroid position, we recommend that one either needs to replan with a larger PTV margin to account for the irregularity in their breathing patterns, or make necessary shifts to compensate for the changes in the target centroid position, if the cone‐beam image does provide the reliable centroid position information for a moving target. For this subject (i.e., the reliability of the centroid position using cone‐beam CT for a moving target), we plan to carry out further studies.

We admit that at the time we performed this study, we did not perform post‐treatment CBCT to verify that the tumor centroid position or overall target volume position had not changed. However, another study has shown that intrafractional tumor position and breathing motion are stable.^(31)^ We later did perform pre‐ and post‐treatment CBCT on other patients and found that the tumor with its motion was not changed. The data have not yet been published.

We not only studied the overall target repositional uncertainty, but also examined the magnitude of the uncertainties in relation to the tumor locations. It was observed that the largest target repositional uncertainties (i.e., the change of target centroid position) are associated with the tumors moving in the S–I direction for the tumors in the LLL, and in the A–P direction for the tumors in the LUL. This observation also concurs with the results reported by Matsugi et al.,^(26)^ where they found that tumors at the lower lobe showed a larger interfraction variation in the GTV position along the craniocaudal direction. In addition, tumors in the RUL tend to have a larger change of the centroid position in the A–P direction than tumors in any other location, and significantly larger in the Lat direction, compared to the tumors in the LUL.

In this work, we also accumulated setup error information. With imaging guidance, this information may not be meaningful. However, the data presented here on setup errors, combined with target repositional accuracy information, could be used for guidance in designing a PTV margin, if no imaging guidance is used. For example, to achieve the 95% confidence level, one needs to use a margin as large as two standard deviations. Since we found the standard deviations for the setup errors to be 4.3 mm in A–P, 4.9 mm in Lat, and 4.9 mm in S–I directions, respectively, and the average target repositional accuracy is 0 in all directions with corresponding SD of 2.5 mm in A–P, 2.4 mm in Lat, and 2.8 mm in S–I directions, we can expect the PTV margin to be in the 9–10mm range, provided that the average errors are added linearly while the SD are added in quadrature.^(42)^

Furthermore, the setup errors in relation to the tumor location were examined in this study, as well. This was achieved by sorting the setup errors according to the tumor locations. It was observed that the setup errors along the S–I direction appeared more pronounced for the tumors in the left lower lobe (LLL) than in other locations. Also, the setup errors for the tumors in the RUL along the A–P direction are slightly larger than tumors in other locations. Except for these observations, we did not find any pronounced correlation between the setup errors and the tumor locations.

## V. CONCLUSIONS

Although the target repositional uncertainty could be as large as 1.7 cm in axial direction, the probability of having a ≥6mm uncertainty is small, in the range of 0.6% to 1.7%. This demonstrates that the PTV margin that is designed based on the ITV outlined on MIP images is appropriate to account for the interfractional tumor centroid positional change, in addition to the residual setup errors, with the use of CBCT imaging guidance. It also implies that the target repositional accuracy is satisfactory following setup error correction. The probability for additional shifts is small (within 2%).
